# The digital legacy in end-of-life care: unspectacular and meaningless, or not enough recognized? An online survey on the attitudes and personal experiences of professionals and volunteers

**DOI:** 10.1186/s12904-026-02212-y

**Published:** 2026-07-01

**Authors:** Anne Meißner, Dafina Mahaj

**Affiliations:** 1 Institute of Business Administration and Business Informatics, Head of IT for the Caring Society Research Group, University of Hildesheim, Hildesheim, Germany; 2 Institute of Social and Organizational Pedagogy, IT for the Caring Society Research Group, University of Hildesheim, Hildesheim, Germany

**Keywords:** Digital Legacy, Attitudes and Experiences, Professional and Volunteer Perspectives, End-of-Life Care, Digital Skills

## Abstract

**Title:**

The digital legacy in end-of-life care: Unspectacular and meaningless, or not enough recognized? An online survey on the attitudes and personal experiences of professionals and volunteers.

**Background:**

The digital era has transformed the way individuals construct and perpetuate their identities, leaving an indelible mark not only in the analogue but also in the digital realm. The digital legacy, comprising online artefacts and virtual traces, plays a pivotal role in shaping one's personality and life narrative. Recognizing its impact on the bereaved and the dying and understanding the nuances of digital legacy are crucial for providing meaningful end-of-life care. Despite the increasing importance of this phenomenon, there is a substantial research gap, and a comprehensive discussion regarding the relevance of digital legacy for dignified and value-oriented end-of-life support is lacking.

**Methods:**

Based on the recommendations of the German Federal Ministry of Justice regarding the handling of digital legacy, a 34-item questionnaire was developed. A nationwide online survey was conducted using a convenience sample. Participants were recruited through palliative and hospice organizations with the aim of capturing attitudes, perceived needs, competency requirements, personal experiences and assessments of various professional and occupational groups, including nursing, medicine, social work, and volunteering. The collected data were analysed using descriptive statistics, and four free-text options integrated into the survey were evaluated through content analysis.

**Results:**

A total of 396 people participated in the survey, mainly professionals (42.3%) and volunteers (30.8%). For approximately one-quarter of the respondents (26.9%), clear identification was not possible, and they made use of the option 'other'. The majority of respondents were female (82.3%), over 50 years old (74.5%) and had > 5 years of professional or work experience (75.7%), predominantly in outpatient care (66.0%). The study showed that the majority of respondents considered the relevance of the digital legacy as high. Moreover, according to 85.8% of the respondents, it does not receive enough attention in end-of-life care. The way in which digital legacies are dealt with varied considerably and were ambivalent in some cases. There is uncertainty with regard to professional responsibilities and the need for guidance, although most respondents perceived their own skills as inadequate and sought to broaden or deepen their knowledge (90.1%). Additionally, there was little engagement with one's own digital legacy. The free-text responses illustrated a lack of awareness, uncertainty and the desire for further training and supporting materials.

**Conclusions:**

The discrepancy between the recognized importance and the lack of direct engagement with one's own digital legacy, as well as the uncertainties in supporting people at the end of their lives, becomes clear and underscores the need for specific programmes that close existing knowledge gaps and promote awareness of how to handle the digital legacy in a valuable and dignified manner in the context of end-of-life care.

**Supplementary Information:**

The online version contains supplementary material available at 10.1186/s12904-026-02212-y.

## Background

Ongoing digitalization has profoundly reshaped various aspects of daily living —including social media, cloud storage, email, and messaging applications— are now inextricably linked to personal routines and social interactions [1]. Over the course of a person’s lifetime, these technologies generate an ever-increasing collection of digital traces, many of which endure long after death [1].

Digital legacy constitutes a distinct aspect within the broader legacy concept [2]. The concept of legacy —or the desire to leave something of enduring value— assumes particular relevance in palliative care. As highlighted in the concept analysis by Timóteo et al. [3], legacy in palliative care encompasses not only tangible assets, but also memories, values, and life experiences intended to transcend death. This process reflects a universal human need for continuity, dignity, and relational connection at the end of life.

Digital legacy, in turn, encompasses the entirety of a person's digital assets, including for example, banking accounts, photos, videos, and social media profiles [4]. Recent conceptual work frames digital legacy as a multidimensional, dynamic, and relational phenomenon. It includes not only consciously curated artifacts but also the unintentional accumulation of digital traces through routine online activities. These digital legacies are shaped both by individual purposefulness and by how meaning is ascribed within familial and social contexts. Managing digital legacy at the end of life therefore involves navigating not only technical and legal issues, but also profound psychosocial and ethical questions regarding identity, memory, and intergenerational continuity [2]. Therefore, for individuals at the end of life, as well as for their families and healthcare providers, this persistent digital data presents both unique opportunities and complex challenges, shaping the psychosocial, cultural, and practical landscape of palliative care.

While numerous studies have explored legacy-making in palliative care [5–19], only few studies have focused on the handling of digital traces during life and the implications of legacy after death for bereaved families. Legacy-making addresses intentional acts to be remembered by others [8], but legacy-making represents only one facet of the broader phenomenon of digital legacy [2]. To date, very little studies have specifically examined digital legacy management in palliative care. For example, a survey among primarily nurses, social care workers, and physicians in ten UK hospices found that conversations about digital legacy are usually initiated by patients, highlighting a lack of proactive engagement and limited professional competence in supporting these processes [12]. Qualitative research has further pointed to the perceived importance of managing digital alongside physical belongings, the necessity of education to improve provider competence, and calls for explicit integration of digital legacy into advance care planning [20]. Additionally, digital legacy has been named as a key research and practice priority in digital health within palliative care [21].

Given the profound implications of digital identity, ongoing digitalization necessitates an interdisciplinary approach to the issues of digital identity construction and digital legacy. Various disciplines, such as social pedagogy, media socialization, ethics, and law, intensively address the challenges and opportunities presented by digital identities [22–26]. Digitally mediated grieving also creates new forms and challenges for bereavement work. Managing digital identities in life and after death requires preserving continuity and authenticity while upholding dignity and privacy, making digital legacy a concern not only for individuals, but also for families and society as a whole.

Despite its growing importance, the topic of digital legacy remains insufficiently addressed in healthcare and social services. There is a lack of clarity regarding the relevance of this issue and systematic support mechanisms. Recognizing its impact on the bereaved and the dying and understanding the nuances of digital legacy are crucial for providing meaningful end-of-life care.

## Aim

The aim of this study was to explore the attitudes, perceived needs, competency requirements, and personal experiences and assessments of various professional and occupational groups in palliative care and hospice work, including nursing, medicine, social work, and volunteering, in relation to the management and significance of digital legacy.

By addressing this substantial research gap and fostering a comprehensive discussion on the relevance of digital legacy for dignified and value-oriented end-of-life support in Germany, this study aims to create a much-needed dialogue and pave the way for future research and policy development in this vital area.

## Methods

### Study design

The study was conducted as a cross-sectional online survey. The research team consisted of researchers with expertise in nursing practice and nursing research (AME) and social pedagogy (DMA).

### Questionnaire development

The survey instrument was an investigator-developed brief questionnaire created according to the usual rules of questionnaire construction [27]. A 34-item questionnaire was developed based on the recommendations of the German Federal Ministry of Justice regarding the handling of digital legacy [28], supplemented by additional questions. It encompasses four subcategories: awareness and attitudes, education needs and competency requirements, experiences and assessments, and demographic data. It was peer-validated for sense-checking and usability by its collaboration partner, the State Hub for Hospice Work and Palliative Care Lower Saxony (LSHPN).

### Sampling strategy and data collection

The survey was conducted using the Lime Survey platform (https://www.limesurvey.org/de). The online survey was open for two months from December 2023 to February 2024 after ethical permission was granted for the study in November 2023. A convenience sample was utilized. An introductory letter explaining the purpose of the survey, along with the survey link, was emailed to all state-specific palliative and hospice organizations in Germany. The state-specific palliative and hospice organisations informed their members (e.g. nurses, social workers, doctors, volunteers) about the survey. Participation was optional.

### Analysis and synthesis

The collected data were analysed using descriptive statistics [29], and the responses to the four free-text questions integrated into the survey were evaluated through content analysis [30].

## Results

For all variables, cases with missing data were excluded from the respective analyses; the valid sample size (n) for each variable is reported in the tables and may therefore differ.

### Participant demographics

A total of 396 individuals participated in the survey. Analysis of the sociodemographic data indicated that 42.3% of the participants were professionals, 19.1% were nurses, 18.8% were social care workers, and 4.4% were medical doctors. Additionally, 30.8% were engaged in voluntary work. Another 26.9% selected the 'other' category, making clear identification of their occupational status not possible. The proportion of female respondents was 82.3%. Furthermore, 74.5% of the participants were over 50 years of age. Regarding professional experience, 75.7% of the participants had over five years of experience in their field. A majority, 66%, were employed in outpatient care. The detailed characteristics of the study participants are presented in Table 1.

**Table 1:** Participant demographics

Gender			male (%)	female (%)	not specified (%)	total (%)	total (n)
			16.9	82.3	.8	100	390
Age	20–29	30–39	40–49	50–59	60 +	total (%)	total (n)
	1.5	7.4	16.5	33.8	40.7	100	393
Years of professional or work experience			< 1 Year	1–5 Years	> 5 Years	total (%)	total (n)
			3.8	20.5	75.7	100	391
Employment field	Nursing	Medicine	Social	Work	Volunteers	Other total (%)	total (n)
	19.1	4.4	18.8	30.8	26.9	100	383
Work Setting			Outpatient hospice and palliative care	Inpatient hospice and palliative care	Other	total (%)	total (n)
			66.0	21.2	12.7	100	377

In the following sections, we will present the findings categorized under the four sub-categories.

### Awareness and professional attitudes

The Awareness and professional attitudes subcategory consisted of nine questions designed as a five-point Likert scale. Approximately one-quarter (24.6%) of respondents reported that they very often, often, or sometimes encounter situations in their work where the digital legacy of a person they are assisting plays a role (not shown in Table 2). Similarly, nearly a quarter (23.3%) agreed that the management of digital legacy is important in their professional practice. A significant majority (83.7%) emphasized the importance of educating patients and their relatives about managing digital legacy, and over half (59.6%) noted its impact on the grieving process of the bereaved. In contrast, only a small percentage (4.1%) believed that digital legacy is adequately addressed in current health and social care, and even fewer (1.1%) believed that it receives sufficient attention in end-of-life care. More details are provided in Table 2.

**Table 2:** Awareness and attitudes

	strongly agree (%)	agree (%)	neutral (%)	disagree (%)	strongly disagree (%)	total (%)	total (n)
The management of digital legacy plays an important role in my work	10	13.3	13.3	30.9	32.5	100	391
It is important to inform patients and their relatives about the necessity of managing digital legacy	48.7	35.0	10.7	3.8	1.8	100	394
Digital legacy affects the grieving process of the bereaved	27.9	31.7	28.6	9.7	2.0	100	391
Digital legacy receives sufficient attention in current health and social care	2.6	1.5	18.2	47.1	30.7	100	391
Interdisciplinary collaboration in the counselling and management of digital legacy is important	43.6	37.4	13.6	4.6	0.8	100	390
Digital legacy already receives sufficient attention in current end-of-life care	0.3	0.8	13.2	51.0	34.8	100	394

The perception of the importance of one's professional or occupational group in managing digital legacies varied significantly. Nursing and social work attributed similarly high importance to their professions, with slightly more than four in ten respondents considering their profession to play a crucial role. The percentage of volunteers was slightly lower and slightly more than three in ten (33.9%), while medical colleagues were the least likely to consider this topic relevant to them. However, it is important to note that the sample size of medical professionals was very small (*n* = 17). Table 3 shows the comparisons.

**Table 3:** Importance of professional groups in managing the digital legacy of patients

	strongly agree (%)	agree (%)	neutral (%)	disagree (%)	strongly disagree (%)	total (%)	total (n)
My professional group plays an important role in managing the digital legacy of patients							
Nursing	12.3	31.5	13.7	30.1	12.3	100	73
Medicine	0	23.5	17.6	41.2	17.6	100	17
Social Work	12.5	29.2	18.1	30.6	9.7	100	72
Volunteers	9.3	24.6	17.8	39.8	8.5	100	118
Other	12.6	28.2	19.4	30.1	9.7	100	103

Even though the surveyed medical professionals were the least likely among the respondents to believe that the topic will play a significant role in their field in the future, more than half still considered it important. In the other two professions, there were even higher approval ratings, ranging from 59.7% (social work) to 65.3% (nursing). These results highlight the differing assessments of the importance of managing digital legacies across professional fields and suggest that an increased negotiation of responsibilities will be essential in the future (Table 4).

**Table 4:** Importance of digital legacy according to professional field

	strongly agree (%)	agree (%)	neutral (%)	disagree (%)	strongly disagree (%)	total (%)	total (n)
Digital legacy will gain importance in my field in the future							
Nursing	27.8	37.5	26.4	6.9	1.4	100	72
Medicine	23.5	29.4	41.2	5.9	0	100	17
Social Work	23.6	36.1	20.8	16.7	2.8	100	72
Volunteers	30.8	44.4	16.2	7.7	0.9	100	117
Other	25.0	46.0	22.0	4.0	3.0	100	100

### Education needs and competency requirements

The subcategory comprised seven questions addressing participants' awareness and attitudes towards digital legacy and its significance for dignified end-of-life care. Five questions were designed as Likert scales, while two included free-text options.

Only approximately one in ten respondents reported having sufficient legal knowledge in this area (10.4%). Slightly more respondents (15%) reported having adequate knowledge of the connection between digital legacy and dignified end-of-life care. Remarkably, 90.1% of the respondents expressed a desire to deepen or expand their knowledge and skills regarding digital legacy. Conversely, only 10.5% felt confident in advising relatives and patients on this topic (Table 5).

**Table 5:** Education needs and competency requirements

	strongly agree (%)	agree (%)	neutral (%)	disagree (%)	strongly disagree (%)	total (%)	total (n)
I have sufficient knowledge of the legal aspects of digital legacy in end-of-life care	3.1	7.3	4.9	40.7	44.0	100	386
I have sufficient knowledge about the connection between digital legacy and providing dignified end-of-life care	5.3	9.7	12.5	44.5	28.0	100	393
I would like to expand or deepen my skills or knowledge regarding digital legacy	55.5	34.6	6.4	2.5	1.0	100	393
I feel confident in advising relatives or patients on the topic of digital legacy	2.3	8.2	6.1	36.7	46.7	100	392
There is sufficient info-material and resources on the topic of digital legacy for supporting people at the end of life	0.5	2.8	34.1	38.7	23.8	100	390

### Specific challenges or uncertainties when dealing with the digital legacy of patients

The qualitative content analysis [19] of the responses to the question, "Are there specific challenges or uncertainties you experience when dealing with the digital legacy of patients? If so, what are they?" revealed several key themes. Notably, the free-text option was used by many respondents (*n* = 252).

There was a significant lack of awareness and perception regarding digital legacies, with many respondents indicating that the topic is not within their awareness, they have no relevant experiences, and there is a general lack of information. All following quotes were translated into English by the authors with the help of ChatGPT4o.

“A feeling of insecurity on both sides, of not knowing enough and of powerlessness in relation to the machinations and effects of the new media. The feeling of being overwhelmed as to where to start in a meaningful way.” (C200/Z296)

Also, a lack of knowledge regarding technical aspects, such as how to effectively delete digital data or who actually has access, are prominent concerns.

"Can you have online accounts deleted without having the password?" (C200/Z18)

The unclear legal situation and lack of actionable options create significant barriers. Legal uncertainties, unclear regulations, and uncertainty about legal responsibilities were commonly mentioned. Many respondents feel inadequately qualified and lack knowledge about the legal aspects of managing digital legacies.

“I'm not truly familiar with the legal side of things, nor do I know where and how I can read up on this topic in a professional way so that I can inform people in my work about this topic at a low threshold.” (C200/Z146)

Finally, there was significant uncertainty about the boundaries of professional responsibilities in providing advice on digital legacies. Professionals were unsure about the extent of their roles and duties.

“What is my role as a caregiver? To what extent is it my job to look after the estate? You can point out that it would be good to have the passwords, etc., but more? Perhaps more about grief counselling after the death, which is a part of palliative work, but only a subordinate one.” (C200/Z55)

Consequently, this results in insufficient guidance and support, exacerbating the challenges in digital legacy management. Additionally, these themes underscore the diverse and multifaceted challenges faced by professionals and volunteers in managing the digital legacies of patients. They highlight the urgent need for greater awareness, enhanced digital skills, clearer legal frameworks, and well-defined professional guidelines to effectively address these issues.

### Requested support

The following results summarize the responses to the question, "What measures or support would you wish from your employer or at the political level to improve the management of digital legacy in end-of-life care?" This analysis highlights the key areas where professionals seek assistance and improvement. It is noteworthy that the free-text option was used by many respondents (*n* = 218).

The primary theme was the need for comprehensive informational and educational materials and training. The respondents expressed a desire for explanatory materials and videos, awareness-raising initiatives, and educational offerings. They emphasized the importance of self-help guides, continuing education opportunities, and practical training sessions and workshops. Additionally, there was a strong call for legal and ethical counselling, clear regulations, and guidelines, as well as public sensitization and outreach efforts. Measures at the employer and political levels, such as regional contacts and institutional support, were also highlighted as crucial for improving the management of digital legacies in end-of-life care.

“More information and guidelines” (C210/Z221)

The feedback highlights the need for comprehensive educational resources, legal and ethical support, public awareness campaigns, and institutional support to effectively address these challenges.

### Experiences and personal assessments

This subcategory comprised 12 questions that provide insights into how respondents manage their own digital legacy and about their perceptions of their own responsibilities in this regard. This assumes that one must first engage with one’s own digital legacy before being able to effectively advise others on the topic.

Approximately six in ten participants had already considered their own digital legacy, at least partially (65.1%), and slightly fewer, exactly a half, had at least partially informed themselves on the matter (50%). At the same time, less than half (43.6%) had a comprehensive or partial overview of their digital accounts, usernames, and passwords. Even fewer have formulated instructions for the event of their death (22.8%) or have created a will for their digital legacy (20.6%), either completely or partially. However, 40.8% of respondents informed a close person about the location of such a list.

A total of 36.3% of the respondents trusted their relatives for their online life after death, while 35% informed a trusted person about their preferences. Interestingly, 59.7% of respondents wished for their online life to be deleted after death, while just under one in ten wanted to delete their online life themselves (8.1%) before they died. Despite these intentions, most respondents had not taken concrete actions thus far and simultaneously planned to manage their own digital legacy in the future (69.3%). The details are shown in Table 6.

**Table 6:** Experiences and personal assessments

	yes (%)	partially (%)	no (%)	total (%)	total (n)
I have personally thought about my own digital legacy	30.9	34.2	34.9	100	392
I have informed myself about digital legacy	13.8	36.2	50.0	100	392
I have already created a will specifying what should happen to my own digital legacy	8.4	12.2	79.3	100	392
I have created a list that includes the names of all my online accounts along with my login details	19.1	24.5	56.4	100	392
I have formulated instructions on what to do with the list in the event of my death	8.7	14.1	77.2	100	391
I have informed close individuals about the location of these documents related to my digital legacy	24.9	15.9	59.2	100	390
I have informed close individuals about my wishes for managing my digital legacy after my death	13.1	21.9	65.0	100	389

	yes (%)	not sure (%)	no (%)	total (%)	total (n)
If you have not planned yet, do you plan to manage your personal digital legacy in the future?	69.3	4.5	26.3	100	358
My online presence (digital legacy) should be deleted after my death	59.7	9.2	31.0	100	390
I will delete my online presence (digital legacy) myself before I die	8.1	19.5	72.5	100	385
My relatives should take care of my online presence (digital legacy) after my death	36.3	24.2	39.4	100	388
I do not care about my online presence (digital legacy) after my death	8.2	76.5	15.3	100	391

### Additional aspects

The final responses to the question "Which additional aspects or personal experiences regarding digital legacy would you like to share with us?" were subjected to qualitative content analysis. Again, the free-text option was used by many respondents (*n* = 322).

Many shared their experiences and thoughts, which further clarified the specific challenges or uncertainties and the resulting requested support. Overall, no new aspects emerged, but personal explanations revealed significant uncertainty, once again highlighting the importance of these survey.

## Discussion

By surveying 396 participants across diverse occupational groups, the study offers critical insights into current practices, role perceptions, structural and educational gaps, as well as personal experiences in dealing with digital legacy.

### Current practices and role ambiguities

A central finding of the study is the contrast between the high perceived relevance of digital legacy and the simultaneously low level of active integration of this issue into palliative care practice—both with regard to patients and to the personal experience of professionals. Awareness and professional attitudes toward digital legacy vary significantly between different occupational groups. While a quarter of the respondents recognized the importance of managing digital legacies in their professional practice, a substantial majority (83.7%) emphasized the need to educate patients and their families on this topic. This highlights a significant gap in current practices, as only a small percentage (4.1%) believe that digital legacies are adequately addressed in health and social care settings.

The varying perceptions of importance among different professional groups, particularly the lower recognition among medical professionals, underscore the need for an integrated approach. Despite the smaller sample size of medical professionals, the finding that more than half of medical professionals still see the importance of digital legacy management suggests the potential for greater engagement if properly supported.

Of particular note is that slightly more than three of four volunteers expect digital legacy to become significant in their field in the future. That is the highest proportion among all respondent groups and underlines the necessity of systematically including volunteers in any consideration of digital legacy aspects in palliative care. Failure to do so risks overlooking essential perspectives and practical resources within palliative care teams.

The findings of this study further illustrate significant ambiguity regarding professional roles and responsibilities in the management of digital legacy. There is a prevailing uncertainty among staff about the extent to which they are responsible for advising and supporting patients and their families. Given the multidimensional challenges involved—which encompass technical, legal, psychosocial, and ethical dimensions—it seems unlikely that any single professional group can adequately address the full complexity of digital legacy in isolation [20, 31]. Therefore, there is an urgent need to clarify roles and responsibilities regarding digital legacy management within palliative care teams. Moreover, our findings highlight the necessity of fostering interdisciplinary collaboration. Notably, in this survey, more than one in eight participants explicitly agreed that interdisciplinary collaboration is essential in this context. Volunteers, given their recognized role and engagement, must be an integral part of these collaborative efforts.

### Structural and educational gaps

Across all groups, there are considerable knowledge gaps and uncertainties regarding various aspects of digital legacy. The low percentage of respondents who were confident in their legal knowledge (10.4%) and their ability to advise on digital legacy (10.5%) points to a critical need for enhanced training and resources. The overwhelming desire among respondents (90.1%) to expand their knowledge and skills underscores the urgency for comprehensive educational programs that are specifically tailored to the practical needs of different professional and volunteer groups. This, again, reflects the demands of previous studies for improved training and resource allocation to better equip professionals for competent advising [12, 20].

### Personal experiences

A key finding of this study is the marked discrepancy between participants limited prior engagement with their own digital legacy and the overwhelmingly high proportion expressing a desire to expand their knowledge in this area. Such gaps are described in the literature as an “awareness–action gap” or “intention–behaviour gap”: in the former, knowledge and problem awareness are lacking, whereas in the latter, there is a clear intention to act, but this does not translate into concrete behaviour [32].

Population-based studies as well as research in the field of palliative and hospice care already confirm the persistence of these gaps: while awareness of the importance of digital legacy is high in various population groups, this rarely results in systematic planning or routine management practices [20, 33, 34]. Remarkably, even among professionals and volunteers in palliative care—who might be expected to show heightened sensitivity to these issues—the topic of digital legacy has not yet become part of everyday care practice. One reason for this may be the multidimensional and dynamic nature of digital legacy itself [2]. Ultimately, managing digital legacy not only requires addressing technical and legal challenges, but also engaging with profound psychosocial and ethical questions, such as those concerning identity, memory, and intergenerational continuity [2].

This ambivalence is also reflected in the finding that more than half of respondents wish their digital legacy to be deleted after their death, yet report considerable uncertainty with regard to the practical implementation. Only about one third support the idea that relatives should take over the management after death, fewer than one in ten intend to delete their online presence themselves, and nearly three quarters do not have a clear opinion or plan concerning the handling of their digital legacy after death. This tension underscores the significant challenges involved in translating attitudes into concrete action and highlights the need for competencies that support effective digital legacy management.

Finally, these findings raise the important question of whether engagement with one’s own digital legacy among professionals and volunteers should be considered a prerequisite for providing adequate support to patients and families facing similar challenges.

## Limitations

The use of an online survey platform may lead to selection bias, as individuals without internet access or with less familiarity with digital tools might be underrepresented. Additionally, the sampling strategy was based on distributing the survey link via email to state-specific palliative and hospice organizations, which could result in uneven participation across different regions and professional groups. The significant percentage of female and older participants might influence the results, suggesting potential gender and age biases in the findings. Due to the convenience sampling approach, self-selection bias cannot be ruled out. Nonetheless, the findings highlight important trends and offer valuable insights that can inform future research directions.

## Recommendations for action

Given these findings, it is crucial to develop educational and support programs that enhance competencies in managing digital legacies and providing related advice. Specifically, training sessions and informational materials could help reduce uncertainties and provide clarity regarding responsibilities. Additionally, legal and organizational frameworks should be established to make it easier for individuals to manage their digital legacies.

## Future research

In the context of rapid digitalization, further research is essential to better understand patients' needs regarding digital legacy. This includes exploring the specific requirements and preferences of patients for managing their digital legacy at the end of life or identifying that digital legacy may be insignificant for some. Additionally, there is a need to investigate effective support options for patients, healthcare professionals and volunteers, including the development and evaluation of educational programs and resources that enhance competencies in managing digital legacies.

Future research should also examine the psychological impact of digital legacy on both patients and their families, including the effects on grief and bereavement processes. By addressing these research areas, we can develop comprehensive strategies that integrate digital legacy management into palliative care practices, ensuring that this important aspect of modern identity is handled with respect and dignity. However, future studies could benefit from broader and more diverse samples to increase generalizability. This includes exploring gender- and age-specific attitudes and needs regarding digital legacy management, which could inform more tailored educational and support programs. Such an approach will help ensure that the findings are reflective of the broader population and address the unique needs of different demographic groups.

## Conclusion

The results highlight a significant discrepancy between the recognized importance of digital legacy and the lack of direct engagement with it, as well as the uncertainties in supporting people at the end of their lives. This discrepancy underscores the urgent need for specific programs that address existing knowledge gaps and promote awareness of how to manage digital legacy in a valuable and dignified manner, particularly in the context of end-of-life care. Establishing interdisciplinary educational strategies, clear professional responsibilities, and supportive legal and organizational frameworks is essential to integrate digital legacy aspects as a meaningful component of palliative care practice.

## Supplementary Information

Supplementary Material 1: Survey items (German language).

Supplementary Material 2: Survey items (English language).

## Data Availability

The datasets used and analysed in the study presented are available from the corresponding author on reasonable request.
