# Correction: The digital legacy in end-of-life care: unspectacular and meaningless, or not enough recognized? An online survey on the attitudes and personal experiences of professionals and volunteers

**DOI:** 10.1186/s12904-026-02228-4

**Published:** 2026-07-21

**Authors:** Anne Meißner, Dafina Mahaj

**Affiliations:** Institute of Social and Organizational Pedagogy, Faculty of Educational and Social Sciences, University of Hildesheim, Universitätsplatz 1, Hildesheim, 31141 Germany

**Correction: BMC Palliat Care 25, 189 (2026)**

**https://doi.org/10.1186/s12904-026-02212-y**

Following publication of the original article [1], the author reported that an older version of the manuscript was erroneously processed by the production team. The revised version, which incorporated recent scientific developments, was erroneously published by the journal as Supplementary Material 3.

The original article has been updated.
