# Supplementary material for: The digital legacy in end-of-life care: unspectacular and meaningless, or not enough recognized? An online survey on the attitudes and personal experiences of professionals and volunteers
Source: BMC Palliat Care. 2026 Jul 1;25:189. doi: 10.1186/s12904-026-02212-y (PMC13326446; doi:10.1186/s12904-026-02212-y)
Supplement: Supplementary file 1 — Supplementary Material 1: Survey items (German language). [file 12904_2026_2212_MOESM1_ESM.pdf]

Nationale Umfrage zum digitalen Nachlass in Palliative Care (DigiNaP)

Einleitung

Sehr geehrte Damen und Herren,
liebe Kolleginnen und Kollegen,

im Rahmen der stetig fortschreitenden Digitalisierung unserer Gesellschaft gewinnt auch der Umgang mit dem digitalen Nachlass von Menschen am Lebensende zunehmend an Bedeutung. Als Fachkräfte in der Pflege, Sozialarbeit, Medizin und Seelsorge spielen Sie eine wesentliche Rolle in der Begleitung von Menschen in dieser sensiblen Lebensphase. Daher ist Ihr Einblick in das Thema des digitalen Nachlasses besonders wertvoll.

Der Digitale Nachlass bezeichnet alle Spuren und Inhalte, die wir im Internet hinterlassen. Dazu gehören z. B. unsere Profile in den sozialen Medien, E-Mails, Online-Konten und digitale Vermögenswerte wie Fotos oder Dokumente genauso wie Hardware (Smartphone, Tablet, PC). Im Falle des Ablebens einer Person ist dieses Online-Leben, der Digitale Nachlass, zu verwalten – genau wie auch der physische Besitz.

Mit diesem Fragebogen möchten wir Ihre persönlichen Erfahrungen, Ihr Bewusstsein für und Ihre Einstellungen zum digitalen Nachlass erfassen. Zudem interessiert uns, in welchem Umfang Sie Beratungsbedarf sehen und welche Kompetenzen Sie für erforderlich halten, um Angehörige genauso wie Patientinnen und Patienten hierin kompetent unterstützen zu können.

Die Beantwortung der Fragen wird ca. 10 Minuten Ihrer wertvollen Zeit in Anspruch nehmen. Ihre Antworten werden selbstverständlich vertraulich behandelt und ausschließlich für den Zweck dieser Erhebung verwendet. Eine anonymisierte Auswertung der Ergebnisse dient dazu, die Betreuung am Lebensende und die damit verbundenen Bedürfnisse in diesem Zusammenhang besser zu verstehen und zu verbessern.

Wir danken Ihnen herzlich für Ihre Teilnahme und Ihre wertvollen Beiträge zu dieser wichtigen Thematik.
Beste Grüße,
Anne Meißner
(Projektverantwortliche)
Universität Hildesheim

Demografische Daten

1	Geschlecht	Weiblich	Männlich	Inter*/Divers	Anderes	
2	In welchem Bereich sind Sie tätig?	Pflege	Sozialarbeit	Medizin	Seelsorge	Anderes
3	Wie viele Jahre Berufserfahrung haben Sie in diesem Feld?	< 1 Jahr	1 bis 5 Jahre	> 5 Jahre		

4	In welchem Setting arbeiten Sie vorwiegend?	Ambulante Hospiz- und Palliativ-versorgung	Stationäre Hospiz- und Palliativ-versorgung			
**Bewusstsein und Einstellung (beruflich)**						
5	Haben Sie bereits Erfahrungen mit dem digitalen Nachlass in Ihrer beruflichen Praxis gemacht?	Ja	Nein			
6	Wie oft begegnen Ihnen in Ihrer Arbeit Situationen, in denen der digitale Nachlass bezüglich einer von Ihnen begleiteten Person angesprochen wird?	Sehr häufig	Häufig	Manchmal	Selten	Nie
7	Das Management des digitalen Nachlasses in meiner Arbeit spielt eine wichtige Rolle.	Stimme voll zu	Stimme eher zu	Weiß nicht	Stimme eher nicht zu	Stimme gar nicht zu
8	Es ist wichtig, Patientinnen und Patienten und deren Zugehörige über die Notwendigkeit der Regelung des digitalen Nachlasses aufzuklären.	Stimme voll zu	Stimme eher zu	Weiß nicht	Stimme eher nicht zu	Stimme gar nicht zu
9	Der digitale Nachlass beeinflusst die Trauerarbeit der Hinterbliebenen.	Stimme voll zu	Stimme eher zu	Weiß nicht	Stimme eher nicht zu	Stimme gar nicht zu
10	Der digitale Nachlass findet in der aktuellen Gesundheits- und Sozialbetreuung genügend Beachtung.	Stimme voll zu	Stimme eher zu	Weiß nicht	Stimme eher nicht zu	Stimme gar nicht zu
11	Eine interdisziplinäre Zusammenarbeit bei der Beratung und Regelung des digitalen Nachlasses ist wichtig.	Stimme voll zu	Stimme eher zu	Weiß nicht	Stimme eher nicht zu	Stimme gar nicht zu
12	Der digitale Nachlass wird in Zukunft in meinem Arbeitsbereich an Bedeutung gewinnen.	Stimme voll zu	Stimme eher zu	Weiß nicht	Stimme eher nicht zu	Stimme gar nicht zu
13	Der digitale Nachlass findet in der aktuellen Versorgung am Lebensende bereits genügend Beachtung.	Stimme voll zu	Stimme eher zu	Weiß nicht	Stimme eher nicht zu	Stimme gar nicht zu
14	Meine Berufsgruppe spielt eine wichtige Rolle im Hinblick auf den digitalen Nachlass von Patientinnen oder Patienten.	Stimme voll zu	Stimme eher zu	Weiß nicht	Stimme eher nicht zu	Stimme gar nicht zu
**Beratungsbedarf und Kompetenzerfordernisse**						

15	Ich habe ausreichend Kenntnisse über die rechtlichen Aspekte des digitalen Nachlasses in der Begleitung am Lebensende.	Stimme voll zu	Stimme eher zu	Weiß nicht	Stimme eher nicht zu	Stimme gar nicht zu
16	Ich habe ausreichend Kenntnisse über den Zusammenhang des digitalen Nachlasses mit einer wert- und würdevollen Begleitung am Lebensende.	Stimme voll zu	Stimme eher zu	Weiß nicht	Stimme eher nicht zu	Stimme gar nicht zu
17	Meine Fähigkeiten oder Kenntnisse zum digitalen Nachlass würde ich gerne erweitern oder vertiefen.	Stimme voll zu	Stimme eher zu	Weiß nicht	Stimme eher nicht zu	Stimme gar nicht zu
18	Ich fühle mich sicher bei der Beratung von Angehörigen oder Patienten zum Thema digitaler Nachlass.	Stimme voll zu	Stimme eher zu	Weiß nicht	Stimme eher nicht zu	Stimme gar nicht zu
19	Es gibt ausreichend Informationsmaterial und Ressourcen für Fachkräfte zum Thema digitaler Nachlass.	Stimme voll zu	Stimme eher zu	Weiß nicht	Stimme eher nicht zu	Stimme gar nicht zu
20	Gibt es spezielle Herausforderungen oder Unsicherheiten, die Sie im Umgang mit dem digitalen Nachlass von Patienten erleben? Welche sind das?	Freitext				
21	Welche Maßnahmen oder Unterstützungen würden Sie sich von Ihrem Arbeitgeber, Ihrer Arbeitgeberin oder auf politischer Ebene wünschen, um den Umgang mit dem digitalen Nachlass in der Begleitung am Lebensende zu verbessern?	Freitext				
**Erfahrungen und Einschätzungen (privat)**						
22	Ich habe mir schon einmal persönlich Gedanken über meinen eigenen Digitalen Nachlass gemacht.	Ja	Nein	Teilweise		
23	Ich habe mich über den Digitalen Nachlass informiert.	Ja	Nein	Teilweise		

24	Eine letztwillige Verfügung darüber, was mit meinem eigenen digitalen Nachlass geschehen soll, habe ich bereits erstellt.	Ja	Nein	Teilweise		
25	Eine Liste, welche die Namen aller meiner Online-Nutzerkonten mitsamt meinen Zugangsdaten umfasst, habe ich erstellt.	Ja	Nein	Teilweise		
26	Anweisungen, was im Sterbefall mit der Liste zu tun ist, habe ich formuliert.	Ja	Nein	Teilweise		
27	Mir nahestehende Menschen habe ich über den Aufbewahrungsort dieser Dokumente, die meinen digitalen Nachlass betreffen, informiert.	Ja	Nein	Teilweise		
28	Mir nahestehende Menschen habe ich über meine Wünsche zum Umgang mit meinem digitalen Nachlass nach meinem Tod informiert.	Ja	Nein	Teilweise		
29	Planen Sie ihren persönlichen Digitalen Nachlass zukünftig zu regeln?	Ja	Nein	Weiß nicht		
30	Mein Online-Leben (digitaler Nachlass) soll gelöscht werden nach meinem Tod.	Ja	Nein	Weiß nicht		
31	Mein Online-Leben nach meinem Tod (digitaler Nachlass) lösche ich selbst bevor ich sterbe.	Ja	Nein	Weiß nicht		
32	Um mein Online-Leben nach meinem Tod (digitaler Nachlass) sollen sich meine Angehörigen kümmern.	Ja	Nein	Weiß nicht		
33	Mein Online-Leben nach meinem Tod (digitaler Nachlass) ist mir egal nach meinem Tod.	Ja	Nein	Weiß nicht		
**Abschluss**						
34	Welche weiteren Aspekte oder persönliche Erfahrungen zum digitalen Nachlass möchten Sie mit uns teilen?	Freitext				
