# Supplementary material for: The digital legacy in end-of-life care: unspectacular and meaningless, or not enough recognized? An online survey on the attitudes and personal experiences of professionals and volunteers
Source: BMC Palliat Care. 2026 Jul 1;25:189. doi: 10.1186/s12904-026-02212-y (PMC13326446; doi:10.1186/s12904-026-02212-y)
Supplement: Supplementary file 2 — Supplementary Material 2: Survey items (English language). [file 12904_2026_2212_MOESM2_ESM.docx]

| **National Survey on Digital Legacy in Palliative Care (DigiNaP)** | | | | | |  |
| --- | --- | --- | --- | --- | --- | --- |
| **Introduction** | | | | | |  |
| Dear Sir or Madam, Dear colleagues,  In the context of the ongoing digitalization of our society, the management of the digital legacy in Palliative and End-of-Life Care becoming increasingly important. As volunteers or professionals in nursing, social work, medicine, and spiritual care, you play a central role in supporting people during this sensitive stage of life. Therefore, your insights on the topic of digital legacy are particularly valuable.  The digital legacy encompasses all traces and content we leave behind on the internet. This includes, for example, our profiles in social media, emails, online accounts, and digital assets such as photos or documents, as well as hardware (smartphone, tablet, PC). In the event of a person's death, this online life—the digital legacy—must be managed, just like their physical possessions.  With this questionnaire, we would like to capture your personal experiences, awareness, and attitudes regarding digital legacy. We are also interested in the extent to which you see a need for counseling on this topic and which competencies you consider necessary to competently support both relatives and patients.  Answering the questions will take approximately 10 minutes of your valuable time. Your responses will, of course, be treated confidentially and used exclusively for the purpose of this study. An anonymized analysis of the results will help us better understand and improve end-of-life care and the associated needs in this context.  We sincerely thank you for your participation and your valuable contributions to this important topic.  Kind regards, Anne Meißner (Project Lead) University of Hildesheim | | | | | |  |
| **Demographic data** | | | | | |  |
| 1. What is your Gender | Female | Male | Inter*/divers | Other |  | |
| 1. How old are you? | up to 29 years | 30-39 years | 40-49 years | 50-59 years | 60 years and 60+ | |
| 1. In which field are you working? | Nursing | Social Work | Medicine | Spiritual Care | Other | |
| 1. How many years of professional experience do you have in this field? | < 1 year | 1. to 5 years | > 5 years |  |  | |
| 1. In which setting do you primarily work? | Outpatient hospice and palliative care | Inpatient hospice and palliative care |  |  |  | |
| **Awareness and professional Attitudes** | | | | | | |
| 1. How often do you encounter situations in your work in which the digital legacy of a person you are supporting is discussed? | Very frequently | Frequently | Sometimes | Rarely | Never | |
| 1. The management of digital legacy plays an important role in my work. | Strongly Agree | Agree | Don’t know | Disagree | Strongly Disagree | |
| 1. It is important to inform patients and their relatives about the necessity of managing digital legacy. | Strongly Agree | Agree | Don’t know | Disagree | Strongly Disagree | |
| 1. Digital legacy affects the grieving process of the bereaved. | Strongly Agree | Agree | Don’t know | Disagree | Strongly Disagree | |
| 1. Digital legacy already receives sufficient attention in current health and social care. | Strongly Agree | Agree | Don’t know | Disagree | Strongly Disagree | |
| 1. Interdisciplinary collaboration in the counselling and management of digital legacy is important. | Strongly Agree | Agree | Don’t know | Disagree | Strongly Disagree | |
| 1. The digital legacy will become more important in my field of work in the future. | Strongly Agree | Agree | Don’t know | Disagree | Strongly Disagree | |
| 1. Digital legacy already receives sufficient attention in current end-of-life care. | Strongly Agree | Agree | Don’t know | Disagree | Strongly Disagree | |
| 1. My professional group plays an important role regarding the digital legacy of patients. | Strongly Agree | Agree | Don’t know | Disagree | Strongly Disagree | |
| **Education Needs and competency requirements** | | | | | | |
| 1. I have sufficient knowledge of the legal aspects of digital legacy in end-of-life care. | Strongly Agree | Agree | Don’t know | Disagree | Strongly Disagree | |
| 1. I have sufficient knowledge about the connection between digital legacy and providing dignified end-of-life care. | Strongly Agree | Agree | Don’t know | Disagree | Strongly Disagree | |
| 1. I would like to expand or deepen my skills or knowledge regarding digital legacy. | Strongly Agree | Agree | Don’t know | Disagree | Strongly Disagree | |
| 1. I feel confident in advising relatives or patients on the topic of digital legacy. | Strongly Agree | Agree | Don’t know | Disagree | Strongly Disagree | |
| 1. There is sufficient info-material and resources on the topic of digital legacy for supporting people at the end of life. | Strongly Agree | Agree | Don’t know | Disagree | Strongly Disagree | |
| 1. Are there specific challenges or uncertainties you experience when dealing with the digital legacy of patients? If so, what are they? | Open text field |  |  |  |  | |
| 1. What measures or support would you wish from your employer or at the political level to improve the management of digital legacy in End-Of-Life Care?? | Open text field |  |  |  |  | |
| **Experiences and Personal Assessments** | | | | | | |
| 1. I have personally thought about my own digital legacy. | Yes | Partially | No |  |  | |
| 1. I have informed myself about digital legacy. | Yes | Partially | No |  |  | |
| 1. I have already created a will specifying what should happen to my own digital legacy. | Yes | Partially | No |  |  | |
| 1. I have created a list that includes the names of all my online accounts along with my login details. | Yes | Partially | No |  |  | |
| 1. I have formulated instructions on what to do with the list in the event of my death. | Yes | Partially | No |  |  | |
| 1. I have informed close individuals about the location of these documents related to my digital legacy. | Yes | Partially | No |  |  | |
| 1. I have informed close individuals about my wishes for managing my digital legacy after my death. | Yes | Partially | No |  |  | |
| 1. If you have not planned yet, do you plan to manage your personal digital legacy in the future? | Yes | Not Sure | No |  |  | |
| 1. My online presence (digital legacy) should be deleted after my death. | Yes | Not Sure | No |  |  | |
| 1. I will delete my online presence (digital legacy) myself before I die. | Yes | Not Sure | No |  |  | |
| 1. My relatives should take care of my online presence (digital legacy) after my death. | Yes | Not Sure | No |  |  | |
| 1. I do not care about my online presence (digital legacy) after my death. | Yes | Not Sure | No |  |  | |
| **Conclusion** | | | | | | |
| 1. What additional aspects or personal experiences regarding digital legacy would you like to share with us? | Open text field |  |  |  |  | |
